# Cases of Gangrene of the Mouth

**Published:** 1840-12

**Authors:** James White


Art. III.—*Cases of Gangrene of the Mouth.* Reported to the Fairfield Medical Association, Ohio, by James White, M. D. Communicated for publication by the Secretary.

This serious disease has attracted less attention than it deserves. It is in vain that the young physician seeks for a satisfactory account of it in our systematic works, and the cases published in our journals are not as numerous as they should be considering the frequency of its occurrence. If it be a secondary instead of a primary affection, it is not on that account the less interesting.

It is not my design to present to the Society an essay on this grave malady, but to read the notes of a few cases which may serve as *data* for its historian. All the cases which have occurred in my own practice, or to which I have been called in consultation, were preceded by constitutional disease, of a febrile kind, such as intermittent and remittent fever, measles, scarlatina, or some other affection. I have thought those fevers which have an epidemic character, more apt to generate it, than those which are purely sporadic. I have not seen it in a child under two years of age, or during lactation. It is peculiarly apt to attack children after protracted fevers and mucous inflammation. The following cases present all the varieties I have met with in practice.

*Case* 1. August, 1823. Called to see Miss G., aged eight years—a fine healthy little girl previous to the present attack. Her mother informed me that she had been ill of fever five days, which was an intermittent of the quotidian type. On examining her mouth I discovered a foul, sloughing ulcer, on the lower lip, one fourth of which appeared scooped out by a process of sloughing, rather than ulceration. The surface of the ulcer was jagged, and covered with a dark, cream colored offensive slough; her gums were sound. The teeth were visible through the ulcer, when the mouth was closed; and there was a considerable discharge of saliva. The progress of this local affection was very alarming. Every time I visited the patient, which was twice a day, a sensible increase of it was visible, not only to myself but also to the family, who beheld it with emotions of grief and horror.

I cannot say how this little girl was treated before I saw her, or whether she had, indeed, any treatment at all. Her physician I suppose gave her no *calomel*, as, at that time, he was preaching against mercury and mercurial doctors.

A variety of topical means were used to arrest the sloughing: diluted muriatic acid, privet tea and alum, camphorated alcohol, a fermenting poultice, and lastly, a carrot poultice, but all to no purpose. The destruction of parts went on, apparently without the least interruption. After having prepared the system well by the employment of mercurial cathartics, the force of the fever being subdued, and a perfect apyrexia established, I commenced the use of tonics—gave bark freely, and Fowler's arsenical solution. The last was given in double doses, for both the mother and sister undertook its administration without each other's knowledge. The consequence was, gastric distress and vomiting ensued, and the tonics were, for the time, discontinued, but she appeared benefitted by the mistake.

With the cessation of fever, the sloughing entirely ceased. The bowels were kept regular, and mild tonics, together with a light unirritating diet perfected the cure. The ulcer, mean while, was poulticed until pretty well filled with granulations; when the poultice was exchanged for a dressing of cerate and lint, and the edges approximated daily. The patient is still living, and, contrary to our anticipations, very little deformity remains.

This case disclosed two important facts:—1st. That the affection of the lip resulted from a peculiar febrile erythism of the system. 2d. That the sloughing did not prove critical or abate the violence of the fever, as is sometimes the case in severe attacks of *herpes labialis*, occurring in the progress of endemic intermittents.

*Case* 2. In the month of June, 1824, I was consulted in the case of Mrs. N.'s child, about three years old. It had been some weeks in ill health, having had slight fever and diarrhœa. The abdomen was very much swollen, and there was considerable emaciation. It was suspected to have worms, and had taken several doses of calomel within the last week. My opinion was asked concerning a soreness of its mouth, which had recently appeared. Upon inspection the mouth was seen to be perfectly sound, excepting that part of the gums which surrounds the last molaris tooth of the upper jaw, on the right side. Here the gum was converted into a grayish white, or cineritious slough, slightly receding from the body of the tooth, which was a little loose, showing its investing membrane to be in a diseased state.

Not having seen a case of the disease which commenced in this manner, I did not anticipate danger, and making a slight prescription sent the patient away. You may form some conception of my feelings when after the lapse of a few days, upon being summoned to visit this unfortunate child, I found all the phenomena of the case changed, and an assemblage of symptoms presented, which but too clearly indicated death or permanent deformity. There was some fever present, the pulse had tolerable strength, the eye was clear, and the intellect not the least disturbed; but the cheek and eyelid of the affected side were very much swollen, and not red, but glossy, with a circular gangrenous patch near the centre of the former, an inch in diameter, of a tawny hue, soft, and slightly depressed. Other parts of the cheek were firmer, showing a low grade of inflammation, with infiltration into the celluar tissue. The breath was highly offensive, and on examining the mouth a dark slough covered the inside of the cheek opposite the gangrenous part without; the gums were black, and the mortification extended high up towards the orbit of the eye. Caries had seized upon the jaw—the teeth were loose—and some of them were plucked out by the patient.

The farther history of this case need not be told. Suffice it to say, that it travelled through all its stages, with the uniformity which distinguishes its career, when not opportunely arrested by the employment of appropriate means. The little patient was perfectly sensible throughout the whole course, and took nourishment as long as she was capable of swallowing.

*Case* 3. In the fall of 1828, Mr. J. S., aged about twenty years, a farmer, in which business he was engaged up to the time of his illness, was attacked with fever and *anasarca*, from which he speedily recovered by the employment of mild antiphlogistic remedies—and continued in good health till January 7th, 1830, at which time the measles became epidemic. He was taken with chilliness and fever; pain in the head, back and limbs; his tongue was coated and its edges red; but there was no catarrh, or any other symptom of the epidemic. He was treated with venesection and purgative doses of calomel and jalap, and calomel followed by castor oil; and when the fever declined, a blister was applied to the nape of the neck, to relieve him of the pain in his head. There was, however, in this case a good deal of drowsiness and hebetude, with some dilatation of the pupils which, together with pain, led me to apprehend mischief within the head. Visiting him on the eighth day of the fever, I found him in the same state of stupor and listlessness, but the pulse was softer, and there was less fever. I discovered some fœtor of the breath, but the gums did not appear to be affected. I believed that a mercurial effect was about being developed. A dose of oil was prescribed, and the blister repeated.

Visiting this patient some days afterwards, I was surprised to find him sitting up before the fire apparently well, but his face tied up with a handkerchief. Enquiring whether his mouth was sore, his mother replied, that "he was troubled with tooth-ache, and that his breath smelt very bad." On examination I found the left cheek very much swollen, pale and glossy, the eyelid of the same side œdematous and nearly closed, and his breath very fœtid. On examining his mouth, a black slough occupied the inside of the cheek and the gums of the three first molares. The sphacelation extended high up towards the orbit of the eye, and the teeth were loose, showing caries of the bone.

The fatal gangrene had established itself, and yet he appeared otherwise free from disease. His pulse was of good strength, fever slight, could take nourishment, and the whole train of febrile phenomena had disappeared.

The general and local remediate measures confided in, were too weak, and consequently altogether inefficient: they did not even abate, much less arrest the progress of the case which went on to a fatal termination.

*Case* 4. In February, 1834, I was called to see Homer, son of Mr. Salmon Shaw, aged eight years, who was taken with the measles, then prevailing epidemically. He had fever, a plentiful rubeolous efflorescence, dry skin, slight chilliness, headache, and pain in the chest, which were very much aggravated by an incessant dry cough; his tongue was coated, and red at the tip and edges;—and his epigastrium very tender, with considerable tension. His father insisted, that no calomel, or other mercurial medicine should be given if I believed it *possible* to cure him without its use. His objections to mercurials probably originated from what he had seen of their abuse; heightened by the recent death of a child three or four years old, in his vicinity, from gangrene of the mouth, under all the horrible circumstances incident to such cases. We readily agreed to give no mercurial, believing it contraindicated, and for the same reason excluded tartrate of antimony. Blood letting repeated, mild laxatives, a blister to the chest, and diaphoretics were found to be adequate to the cure, which appeared to be pretty well established, when symptoms of gangrene made their appearance.

Happily, not a single grain of calomel had been used in the treatment of this patient; and it affords another evidence that *gangrene* of the mouth is, sometimes at least, not the product of that mineral.

The manifestation of the approach of the gangrene, was the appearance of a superficial slough in the gums covering two of the molar teeth, on the right side of the upper jaw. The gums in other respects appeared sound, and there was no fœtor of the breath, or flow of saliva. I confess that I did not at that time suspect the danger that was impending; but in the course of a few days the symptoms were sufficiently developed to admit of no doubt on that subject. The slough had increased, the periostium of the teeth and alveolar processes had become implicated, the breath was slightly fœtid, and there was some irritative fever. A mild laxative was given, and a light nutritive diet enjoined. A prescription was made to be applied every six hours to arrest the progress of the sloughing. I requested Mr. S. to make a daily record of the progress of the case, and of the effects of the remedies, which is here subjoined:

"*March* 1*st*. Gums eaten out between two teeth—applied prescription. 2d. One tooth loose, eating continued, prescription as above. 3d. Canker spreading very fast, and communicated to the cheek. 4th. A tooth extracted—applied muriatic acid and honey, equal parts, every four hours—canker spreading rapidly. 5th. Muriatic acid and honey continued, swelling of the cheek increased. 6th. The same application continued—the flesh separated from the jaw bone, about half an inch up, and three-fourths of an inch long. Applied charcoal, and continued the acid and honey. 7th. The same application continued—another tooth extracted." 8th. The gangrene now appeared to have made such progress, as to leave no hope in the case. He was in that condition, from which I had never seen a single recovery. The right cheek was very tense, swollen, pallid and glossy; the intumescence extended to the right eye, which was closed, and to the lips, drawing the right commissure to one side, from which the saliva flowed copiously. The whole expression was frightfully distorted, and the breath had its characteristic fœtid odor. Caries had indeed seized upon the jaw, from which the teeth were extracted, and the cheek appeared, internally, hollowed or scooped out, about the size of a fifty cent piece, and was covered with a thick black slough. The mortification appeared to extend from the jaw and cheek, high up towards the orbit of the eye; and judging from the celerity with which the gangrene had progressed, during the last twenty-four hours, it was thought that by the next day it would, in all probability, perforate the cheek. The patient had fever, but the skin was soft and slightly moist, pulse of good strength, intellect clear, and apparently without pain. His appetite was good and he took chicken soup, gruel, panada &c.; his bowels were kept regular by mild laxatives, and enemata. Tonics were not deemed necessary, and no blister was applied to the cheek, from a conviction that it would do no good; indeed, in every case in which I had employed it, a blister had done manifest harm, by subjecting the patient to unnecessary pain, and adding to the general irritation.

Under these discouraging circumstances I examined anew Dr. Jackson's excellent essay on *Gangrenopsis*, published in the Medical Recorder, 1826, vol. XII. Among other valuable precepts, indicated for the management of these bad cases, he makes mention of the Baron Van Swieten's remedy, *undiluted* muriatic acid. His words are, "But Van Swieten used undiluted muriatic acid with great success." He says—"It presently stopped the gangrene, and soon after the eschar has separated from the parts in which there was life—nor have I ever known this application to fail me, except where the gums being entirely putrid, the jaw bone was affected; for then I could not prevent its being carious; but if the soft parts only in the inside of the mouth are the parts that are gangrened, it will certainly cure them." The Baron speaks of his remedy in the strongest terms of commendation, when applied in gangrene affecting the "soft parts only in the inside of the mouth." He seems distrustful of it however, when the bone is carious. I resolved, notwithstanding, to use the strongest muriatic acid; and shall proceed with Mr. Shaw's daily report, which continues thus:

"8th, four o'clock, P. M., applied undiluted muriatic acid; six o'clock same; seven o'clock same; and continued to apply it every hour till 3 o'clock A. M., next morning; mortification arrested, and reaction established; sensibility in the parts acute. 10th. Same application twice a day; the sensibility of the parts acute. 11th to 19th. Applied tinct. myrrh and alum, appearances very favorable."

Here then, we find undiluted muriatic acid, not only capable of arresting mortification of the soft parts within the mouth, but also, that it is equally adequate to the subdual of caries, since we find that on the 9th day, the sphacelation was arrested, both in the bone and soft parts.

A swab was made by rolling and securing with thread, a piece of rag on the end of a stick, which, when charged with the acid, was carried up repeatedly, and brought in contact with every point of the diseased surface. These repeated and fresh applications of acid, appeared to give no uneasiness at first, excepting paroxysms of cough from the passage of muriatic acid gas into the glottis, in inspiration. The acid was, as it were, *kneaded* into the parts. It presently dissolved or decomposed the sloughs, and came into contact with the living parts, which re-acted healthfully under its enlivening stimulation. It was at this crisis only that the patient began to experience pain, which continued to be more and more acute, under each successive application, until he could no longer bear it; which was the case on the eleventh day, when it was discontinued, and tinct. myrrh with solution of alum, substituted.

When the sphacelation ceased the wound soon put on a healthy appearance, granulated and secreted healthy pus. The swelling receded from the cheek, and eyelids; and a proportionate amendment occurred in the system at large; the digestion and health improved, and in a short time the patient was able to sit up. The ulcer, however, did not heal until a portion of the dead bone was extracted from the jaw, about the nineteenth or twentieth day; it then soon filled up, and cicatrized. The deformity is but slight.

*Case* 5. James, son of Mr. C., aged 5 years, was seized with intermittent fever of a quotidian type, in September, 1836. There was nothing in the symptoms to distinguish it from an ordinary endemic intermittent, except that he suffered very much from severe headache, which postponed the time for giving tonics. The treatment consisted of blood letting, three doses of calomel, always speedily worked off by castor oil, and infusion of spigelia by which a few lumbricoides were discharged. A few days after having discontinued our visits, his mother brought him to us, believing he was salivated. Upon examining his mouth, I found the gums sound, with the exception of that around the second molar tooth of the right side of the superior maxillary bone, which was converted into a yellowish white slough, receding from the body and neck of the tooth. The cheek was also affected, lying swollen but not glossy. The mucous surface of the cheek being against the diseased tooth, was somewhat scooped out and sloughy, with elevated, red margins. The tooth was still firm, but caries had nearly destroyed its external lateral surface. It was believed to be a case of incipient canker, or gangrena oris.

The chill and fever still continuing though in a subdued degree, tonics were prescribed, which arrested the paroxysms.

A solution of sulphas cupri and pulv. cinchon., as recommended by Dr. Coates, was applied three times a day, till the sloughy appearance of the part was removed, and the caries of the tooth was arrested, when mild unirritating washes were used to complete the cure.

This case was taken in time. The disease had not extended to the periostium, and the tooth remained firm It differed however from other cases, in which the teeth were not affected, excepting in their invest membrane. They became loose, and were extracted or fell out.

As already intimated I have never seen a case of gangrœna oris, in any form, without its having been preceded by some serious constitutional derangement. Thus, at one time we find it engrafted upon bilious remittent or intermittent fever; at another, on fever with visceral inflammation; at another on measles, or scarlatina. I lost two children of this disease in 1823. The local affection occurred in each, in the first week of the fever, which was a bilious remittent. It may, however, occur in the wane of the disease, or when the system is verging towards collapse, or as a sequela.

The treatment of these cases must have regard, first, to the pathological state of the system at large; and, secondly, to the local affection. The latter is the result of constitutional derangement of a febrile kind: it was especially so in the first reported case. The sloughing could not be arrested until the fever was conquered. Therefore, the removal of the fever, or correcting the general constitutional derangement, is of the first importance; and indeed, an essential preliminary step towards arresting the local disease, in its first stage. The local disease on its appearance cannot modify our prescriptions, or afford contra-indications in regard to the employment of general remedies. Besides the general means instanced above, local measures are of equal, and in some cases of paramount importance. The best we have used to stop the sloughing in its incipient stage, is a saturated solution of *sulphas cupri*, with which the part is to be touched three or four times in the twenty-four hours, until the sloughing is arrested.

If these means do not effect that object, and especially if the mortification have made much progress, having extended to the cheek, which is occupied by a deep, dark slough; and to the bone, which becomes carious, no remedy has, I believe, stronger claims to our confidende, than *undiluted muriatic acid*. It is vain to expect an arrestation of gangrene, by the employment of general remedies at such a crisis, however valuable they may be as adjuvants. Nothing short of the application of a strong local stimulant, such as is capable of making a vigorous change in the organic actions of the part, producing a lively reaction, and at once elevating the parts to a grade of excitement incompatible with the sloughing process, can avail in such a case.

*October*, 1838.

